# Calmodulin mutations affecting Gly114 impair binding to the Na_V_1.5 IQ-domain

**DOI:** 10.3389/fphar.2023.1210140

**Published:** 2023-08-16

**Authors:** Malene Brohus, Ana-Octavia Busuioc, Reinhard Wimmer, Mette Nyegaard, Michael Toft Overgaard

**Affiliations:** ^1^ Department of Chemistry and Bioscience, Aalborg University, Aalborg, Denmark; ^2^ Department of Health Science and Technology, Aalborg University, Gistrup, Denmark

**Keywords:** calmodulin, calmodulinopathy, arrhythmogenic, cardiac ion-channel regulation, calmodulin target binding, experimental variant interpretation, SCN5A, NaV1.5

## Abstract

Missense variants in *CALM* genes encoding the Ca^2+^-binding protein calmodulin (CaM) cause severe cardiac arrhythmias. The disease mechanisms have been attributed to dysregulation of RyR2, for Catecholaminergic Polymorphic Ventricular Tachycardia (CPVT) and/or Ca_V_1.2, for Long-QT Syndrome (LQTS). Recently, a novel *CALM2* variant, G114R, was identified in a mother and two of her four children, all of whom died suddenly while asleep at a young age. The G114R variant impairs closure of Ca_V_1.2 and RyR2, consistent with a CPVT and/or mild LQTS phenotype. However, the children carrying the *CALM2* G114R variant displayed a phenotype commonly observed with variants in Na_V_1.5*,* i.e., Brugada Syndrome (BrS) or LQT3, where death while asleep is a common feature. We therefore hypothesized that the G114R variant specifically would interfere with Na_V_1.5 binding. Here, we demonstrate that CaM binding to the Na_V_1.5 IQ-domain is severely impaired for two CaM variants G114R and G114W. The impact was most severe at low and intermediate Ca^2+^ concentrations (up to 4 µM) resulting in more than a 50-fold reduction in Na_V_1.5 binding affinity, and a smaller 1.5 to 11-fold reduction at high Ca^2+^ concentrations (25–400 µM). In contrast, the arrhythmogenic CaM-N98S variant only induced a 1.5-fold reduction in Na_V_1.5 binding and only at 4 µM Ca^2+^. A non-arrhythmogenic I10T variant in CaM did not impair Na_V_1.5 IQ binding. These data suggest that the interaction between Na_V_1.5 and CaM is decreased with certain CaM variants, which may alter the cardiac sodium current, I_Na_. Overall, these results suggest that the phenotypic spectrum of calmodulinopathies may likely expand to include BrS- and/or LQT3-like traits.

## Introduction

The cytosolic calcium (Ca^2+^) binding protein calmodulin (CaM) serves as a critical mediator of intra-cellular Ca^2+^ signals in a multitude of physiological processes (Chin and Anthony, 2000; Xia and Storm, 2005; Clapham, 2007; Sorensen et al., 2013; Berchtold and Villalobo, 2014). The multifaceted nature of CaM comes from its ubiquitous expression and its ability to interact with, and relay information to, more than 350 cellular target proteins (Yap et al., 2000; Tidow and Nissen, 2013).

**FIGURE 1:**
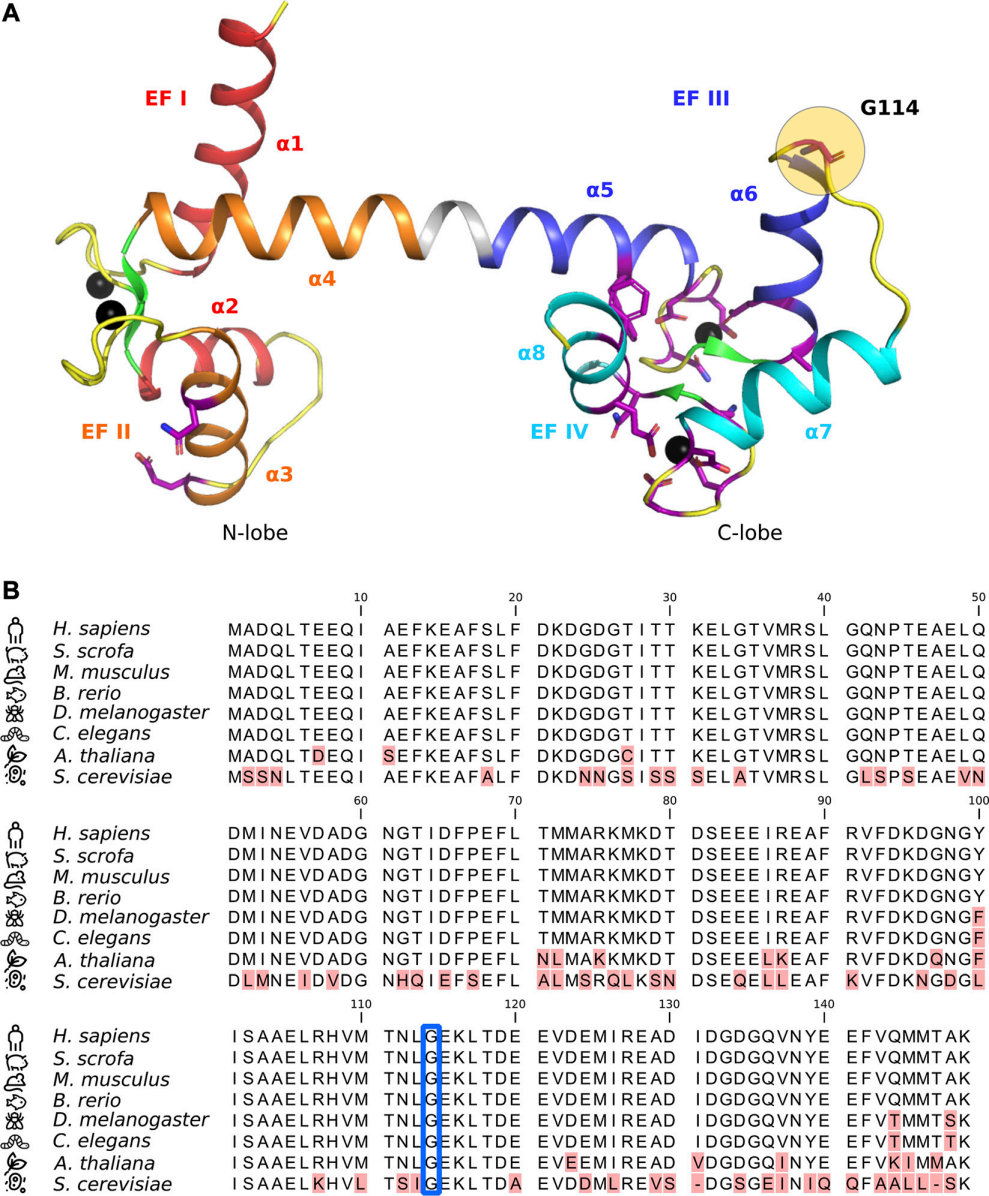
**(A)** Crystal structure of Ca^2+^-bound calmodulin (CaM) (PDB ID: 1CLL) with EF hands I (*red*), II (*orange*), III (*blue*), and IV (*cyan*) indicated along with their corresponding alpha-helices (α1-8). Beta-sheets are shown in green and flexible loops/unstructured regions in yellow. Amino acid residues known to harbor arrhythmogenic substitutions (Crotti et al., 2019) are shown in purple stick representation, with residue G114 highlighted with a yellow circle. Ca^2+^-ions are shown as black spheres. **(B)** CaM protein sequence alignment. Red highlights indicate amino acid differences or gaps in the alignment. Residue G114 is indicated by a blue frame. Numbering is according to immature human CaM (including the initial M).

This extraordinary versatility is due to the two lobes of CaM, the N-lobe and C-lobe, each containing two Ca^2+^-binding EF hands (Figure 1A). The lobes differ in Ca^2+^-affinity and -kinetics, allowing CaM to respond to changes in Ca^2+^ over a broad range of concentration and time. This range is even further expanded by target-specific changes in Ca^2+^ binding-affinities and -kinetics upon CaM binding to protein targets (Villarroel et al., 2014; Søndergaard et al., 2015a; Søndergaard et al., 2015b).

The cellular importance of CaM Ca^2+^-sensing and integrity is highlighted by the protein’s unique genetic architecture and evolutionary conservation. Mammals have three independent genes (*CALM1-3*) that all encode an identical CaM protein. Moreover, the protein sequence is invariant in all vertebrates, underpinning the extreme selection pressure against any amino acid variation in this central Ca^2+^-sensor protein (Figure1B) (Berchtold et al., 1993; Toutenhoofd and Strehler, 2000; Friedberg and Rhoads, 2001).

As a result, genetic variation in all three *CALM* genes is ultra-rare. Until the first human missense variant was discovered in 2012, and linked to a severe cardiac arrhythmia (Catecholaminergic Polymorphic Ventricular Tachycardia (CPVT)) and sudden cardiac death (SCD) (Nyegaard et al., 2012), mutations in the *CALM* genes were considered incompatible with life (Jensen et al., 2018). Since the initial discovery of human missense variants linked to CPVT, mutations have also been identified in individuals affected by long QT syndrome (LQTS) (Crotti et al., 2013) and Idiopathic Ventricular Fibrillation (IVF) (Marsman et al., 2014). In 2019, Crotti and co-workers presented an extensive collection of 28 unique CaM variants, identified in 74 carriers, in the International Calmodulinopathy Registry (Crotti et al., 2019). *CALM* variant carriers in this registry present with cardiac arrhythmia phenotypes including LQTS (49%), CPVT (28%), overlap LQTS/CPVT (4%), and a few cases of IVF, sudden unexplained death (SUD), or atypical phenotypes (Crotti et al., 2019).

Clinical characteristics of the calmodulinopathies include an early age of onset (a mean of 1.5 years for LQTS and 6 years for CPVT) and a high risk of a major arrhythmic event (68%), such as cardiac arrest or SCD/SUD (Crotti et al., 2019). The molecular mechanisms underlying the two main phenotypes has largely been ascribed to specific dysregulation of the two primary cardiac Ca^2+^ channels, Ca_V_1.2 for LQTS, and RyR2 for CPVT-like phenotypes (Limpitikul et al., 2014; Yin et al., 2014; Søndergaard et al., 2019; 2020; 2017; Nyegaard and Overgaard, 2019; Holt et al., 2020). The broad phenotypic spectrum caused by CaM variants, including mechanistically different cardiac arrhythmias, is likely a consequence of CaM serving as a key regulator of multiple cardiac ion-channels, besides Ca_V_1.2 and RyR2, that control cardiac excitation-contraction coupling. Indeed, given the vast number of CaM-regulated proteins, the phenotypic spectrum of calmodulinopathies is likely to expand even further as more carriers are discovered (Jensen et al., 2018; Urrutia et al., 2019).

In 2019, a novel *CALM2* variant, G114R (immature protein numbering including initial Met), was identified in an Australian mother and two of her four children. Over a 10-year period, all four children died suddenly and unexpectedly while asleep, at ages ranging from 19 days to 18 months (Brohus et al., 2021). Further, the two children carrying the G114R variant had infections at the time of death, implying a potential presence of fever. In 2021, we showed that the G114R variant impairs CaM’s ability to bind Ca^2+^-ions and to interact with and regulate Ca_V_1.2 and RyR2, with an impact suggesting an arrhythmogenic potential consistent with CPVT, IVF, or mild LQTS (Brohus et al., 2021).

Death while asleep or at rest have only been observed in a small subset of CaM variant carriers, and mainly for CaM variants with a severe impairment of Ca^2+^ binding and/or Ca^2+^-dependent inactivation of Ca_V_1.2, larger than the effect imposed by G114R (Brohus et al., 2021). Therefore, the phenotype of the children carrying the *CALM2* G114R variant to some degree represents an expansion of the known clinical manifestations of CaM variant carriers. The phenotype more closely resembles that of carriers of missense variants in the cardiac sodium channel, Na_V_1.5, for whom major arrhythmic events or death while asleep is a common feature (Schwartz et al., 2001; Postema and Wilde, 2008; Takigawa et al., 2008). Given that CaM is critical for Na_V_1.5 function, we hypothesized that the G114R variant would specifically interfere with Na_V_1.5 binding.

The Na_V_1.5 channel is implicated in both Brugada Syndrome (BrS) and LQT3, arrhythmic diseases that result from divergent molecular mechanisms. Intriguingly, both phenotypes can be caused by Na_V_1.5 channel mutations that perturb the interaction with and modulation by CaM (Shah et al., 2006; Yan et al., 2017; Urrutia et al., 2019; Kang et al., 2021; Wu and Liang, 2021). In some cases, the same Na_V_1.5 variant causes both phenotypes, which alludes to the difficulty in variant genotype-phenotype interpretation (Supplementary Figure S1).

**FIGURE 2:**
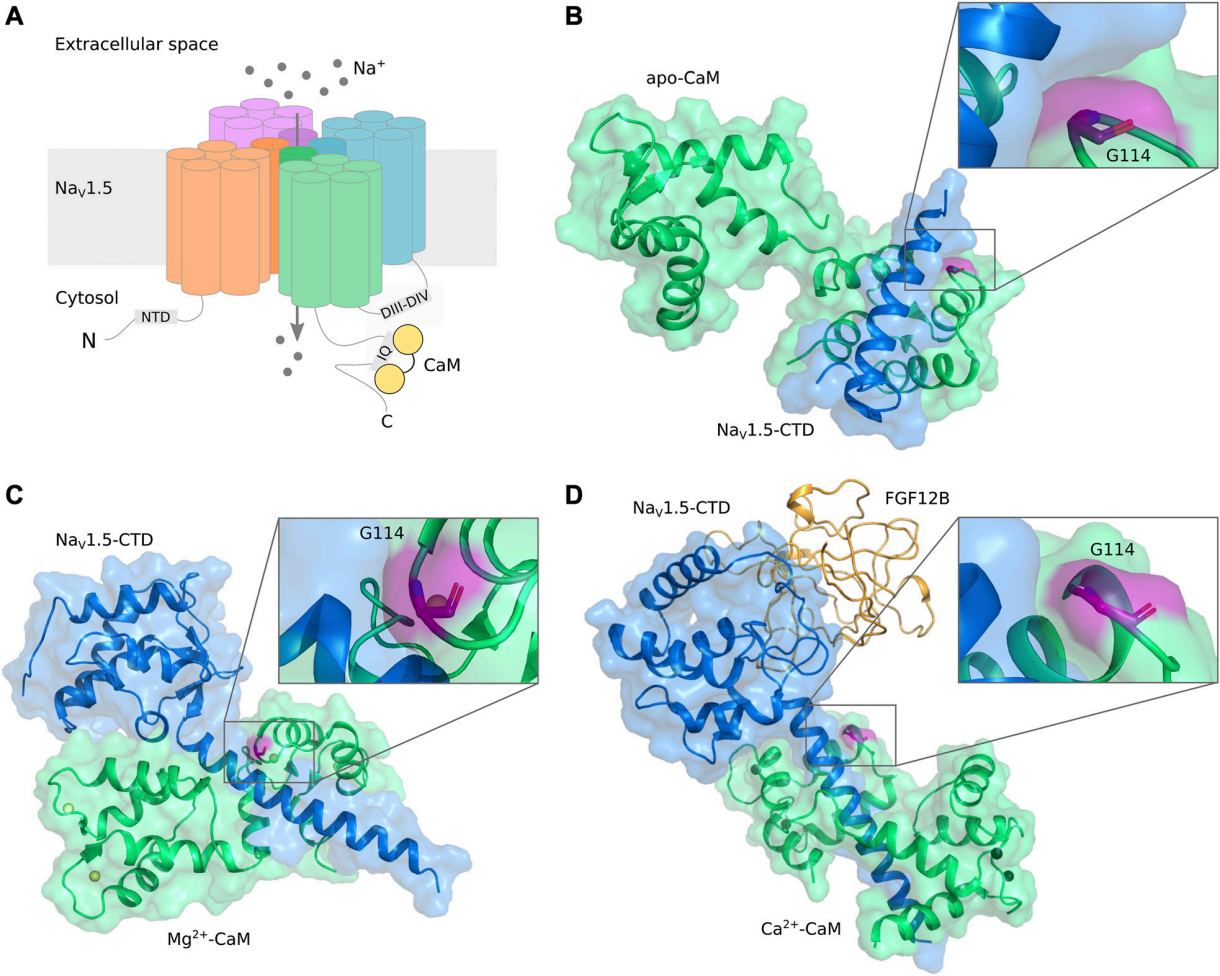
**(A)** Schematic representation of Nav1.5 structural elements and interaction with CaM. **(B–D)** Visualization of CaM-G114 in Na_V_1.5/CaM complexes. CaM (*green*) and the Na_V_1.5 CTD (*blue*) are displayed in cartoon and surface representation with CaM-G114 (*purple*) displayed in stick representation. The insets show that G114 is located at the interface between the CaM EF hand III-IV loop and the Na_V_1.5-IQ domain. **(B)** apo-CaM, PDB ID 2L53 (Chagot and Chazin, 2011); **(C)** Mg^2+^-bound CaM, PDB ID 4OVN (Gabelli et al., 2014); **(D)** Ca^2+^-bound CaM, PDB ID 4JQ0 (Wang et al., 2014). Ca^2+^ (*black*) and Mg^2+^ (*yellow*) ions are shown as spheres. CaM, calmodulin; Na_V_, voltage-gated sodium channel; CTD, C-terminal domain; FGF, fibroblast growth factor (*orange*).

The gating of Na_V_1.5 is modulated by CaM in a bi-directional manner: Both channel activation (peak current), fast inactivation, and persistent current depend on CaM. Several CaM binding domains (CaMBDs) have been identified, but their individual roles in the bi-directional modulation by CaM is still unclear (Kang et al., 2021). While the primary CaM binding site is an IQ-motif located in the C-terminal domain (CTD) of Na_V_1.5 (Chagot and Chazin, 2011; C; Wang et al., 2014; Gabelli et al., 2014; C; Wang et al., 2012; Gabelli et al., 2016), CaM has also been shown to interact with a preIQ-domain in the CTD (Yoder et al., 2019), the “inactivation gate” in the DIII-DIV linker (Potet et al., 2009; Sarhan, Van Petegem, and Ahern, 2009; Sarhan et al., 2012; Johnson et al., 2018), and an N-terminal domain (NTD) (Wang et al., 2020) (Figure2A).

In this study, we illustrate that CaM-G114 is located exactly in the binding interface between CaM and the IQ-domain of Na_V_1.5 and demonstrate that CaM variants G114R and G114W impair the interaction with the IQ-domain in a Ca^2+^-dependent manner with the largest impact occurring at a free Ca^2+^-concentration range of 3 nM–4 µM. Thus, the apoCaM interaction with the Na_V_1.5-IQ domain is impaired for these CaM variants and their Ca^2+^-sensing ability in the CaM/Na_V_1.5-IQ complex has markedly changed.

## Materials and methods

Materials and methods can be found in the Supplementary Material.

## Results

### The CaM-G114 residue is extremely evolutionarily conserved and located at the CaM/Na_V_1.5 IQ-domain interface

CaM-G114 is the terminating residue of the second helix of EF hand III and it so far constitutes the only amino acid residue known to harbor a mutation in the loop between EF hands III and IV (L113-T118) (Figure 1A) (Bycroft et al., 2018; Crotti et al., 2019; Chen et al., 2022). A protein sequence alignment of CaM from different species shows that residue 114 is extremely evolutionarily conserved (Figure1B, *blue square*). It is a glycine in all species investigated, including yeast, emphasizing the universal importance of its integrity.

High resolution structures of CaM (*green*) in complex with the Na_V_1.5-CTD (*blue*) reveal that CaM-G114 (*purple*) is located at the interface between CaM and the channel (Figure 2). In the apo-form, only the C-lobe of CaM binds to the Na_V_1.5 IQ-domain (Figure 2B), whereas in the Mg^2+^- and Ca^2+^-bound forms, CaM wraps around the Na_V_1.5 CTD with both its lobes (Figure 2C, D). In all cases, the interaction brings CaM-G114 and the IQ-domain into close proximity (Figure 2, insets). We therefore hypothesized that substitution of residue G114 would affect the binding between CaM and the Na_V_1.5 channel.

### The G114R and G114W variants impair the interaction between CaM and the Na_V_1.5 IQ-domain

**FIGURE 3:**
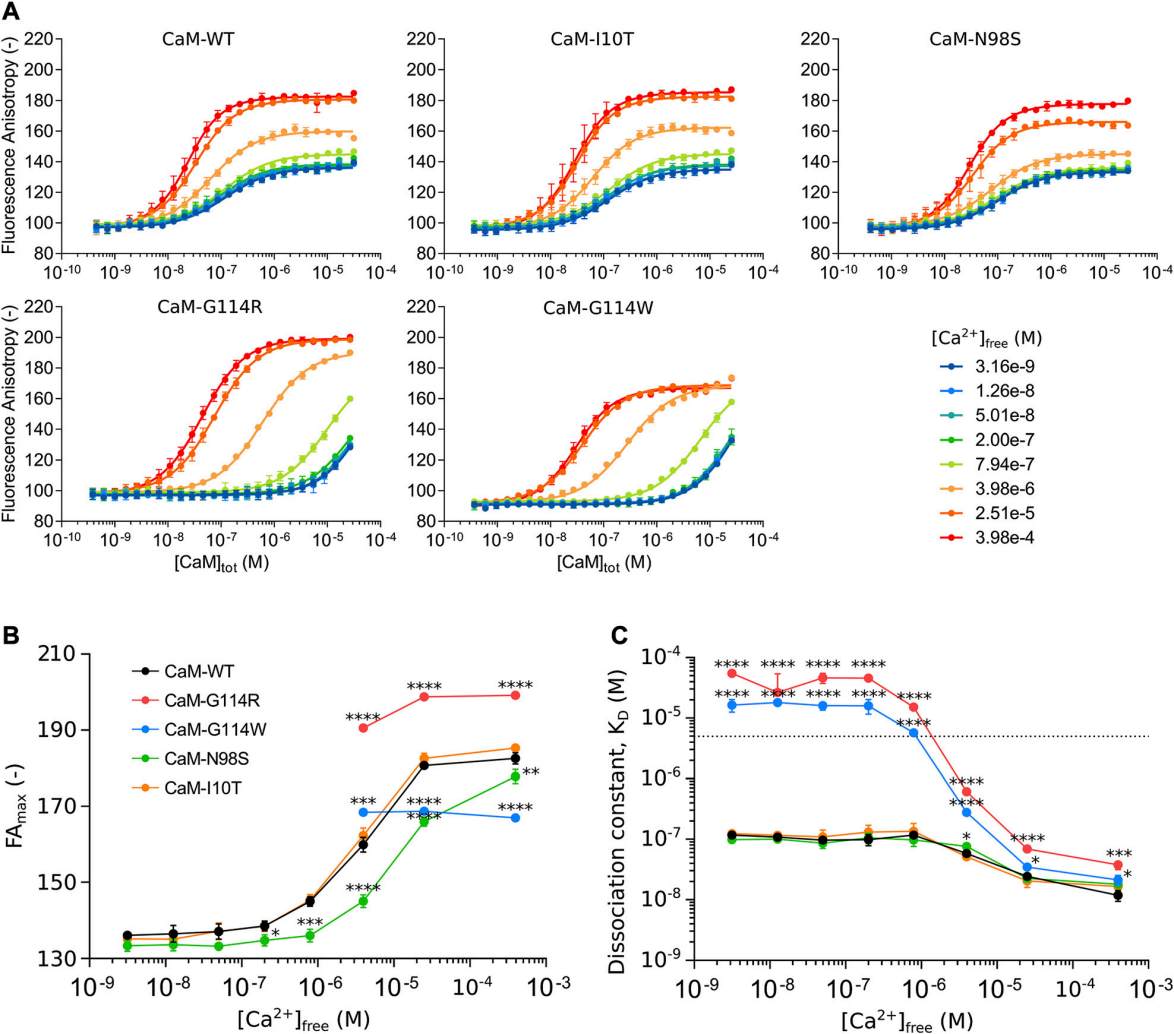
**(A)** CaM binding to the Na_V_1.5 IQ-domain monitored by fluorescence anisotropy (FA) as a function of total CaM-concentration ([CaM]_tot_) at eight free Ca^2+^ concentrations ([Ca^2+^]_free_). **(B)** Ca^2+^-dependent conformational changes of the CaM/Na_V_1.5 IQ-domain complex represented by changes in the maximum FA plateau (FA_max_) as a function of [Ca^2+^]_free_. **(C)** Ca^2+^-dependent changes in the CaM affinity of the Na_V_1.5 IQ-domain represented by the dissociation constant (K_D_) as a function of [Ca^2+^]_free_. Color scheme as in panel B. Each data point represents the mean of three replicates with the standard deviation shown as error bars. For panel B and C, statistically significant differences between CaM-WT and other variants were determined by a 1-way ANOVA at each Ca^2+^-concentration with Dunnett’s multiple comparisons test: **** (*p*-value <0.0001), *** (*p*-value <0.001), * (*p*-value <0.05).

To test the hypothesis that the integrity of CaM-G114 is essential for the interaction with the Na_V_1.5 IQ-domain, we monitored the fluorescence anisotropy (FA) signal of the TAMRA-labeled IQ-domain during titration with CaM at eight different Ca^2+^-concentrations, resulting in eight binding curves for each CaM variant (Figure 3A). The CaM variant N98S was included as an arrhythmogenic control, known to cause both LQTS and CPVT (Nyegaard et al., 2012; Makita et al., 2014; Jiménez-Jáimez et al., 2016). Another CaM variant, CaM-I10T, identified in the UK Biobank resource (Bycroft et al., 2018), was included as a non-arrhythmogenic control.

The interaction between CaM-WT and the Na_V_1.5 IQ-domain depends on the level of Ca^2+^, apparent as a change in FA values for the CaM-saturated IQ-domain from low (Figure 3A, *blue*) to high (Figure 3A, *red*) Ca^2+^ concentrations. The assay thus allowed us to explore the Ca^2+^-dependency of the interaction, by determining the maximum FA plateau (FA_max_) (Figure 3B, Supplementary Table S1) and the binding affinity (Figure 3C, Supplementary Table S2) at each of the eight Ca^2+^-concentrations.

The FA signal is a measure of the tumbling rate of the TAMRA-labeled Na_V_1.5 IQ-domain (Rossi and Taylor, 2011). As more CaM is added, more CaM/Na_V_1.5 IQ-domain complex forms, and the FA signal will increase (due to a reduced tumbling rate of the IQ-domain) until reaching FA_max_ which represents the tumbling rate of the saturated complex (Figure 3A). The tumbling rate depends on the conformation of the complex, and a plot of the FA_max_ value as a function of [Ca^2+^]_free_ reveals a Ca^2+^-dependent increase in FA_max_ of the CaM-WT/Na_V_1.5 IQ-domain complex, in turn reflecting a Ca^2+^-induced conformational change (Figure 3B, *black*, Supplementary Table S1). The Ca^2+^-dependent conformational change is accompanied by a 10-fold increase in the CaM-WT binding affinity of the Na_V_1.5 IQ-domain (Figure 3C, *black*, Supplementary Table S2).

Curiously, the Ca^2+^-dependent development in the FA signal for the CaM-G114 variants is very different from CaM-WT. First, no FA_max_ plateau is reached at free Ca^2+^ concentrations <4 µM (Figure 3A, B). Second, the FA_max_ values ≥4 µM free Ca^2+^ are significantly different from those of the CaM-WT/Na_V_1.5-IQ complex (Figure 3B, *red and blue*, Supplementary Table S1). Interestingly, the change in FA_max_ imposed by the two G114 substitutions occurs in opposite directions relative to CaM-WT at saturating Ca^2+^ (FA_max_ increases for CaM-G114R and decreases for CaM-G114W, Supplementary Table S3).

Moreover, the CaM-G114R and -G114W mutations cause a dramatic reduction in IQ-domain binding affinity compared to CaM-WT (Figure 3C, *red* and *blue*, Supplementary Table S2). At Ca^2+^-concentrations below 4 μM, the affinity is reduced to an extent where the dissociation constant could not be accurately determined (K_D_ < 5 µM) (Figure 3C, *stapled line*). However, the data demonstrates that the affinity is reduced at least 47-fold compared to CaM-WT at these Ca^2+^-concentrations and estimates of K_D_-values could be determined by assuming an identical FA_max_ value at all Ca^2+^ concentrations (Figure 3C, see Methods section for details, Supplementary Table S2, S4). At free Ca^2+^-concentrations at and above 4 μM, the IQ-domain affinity of CaM-G114R and -G114W is still significantly reduced compared to CaM-WT, but to a smaller extent (1.4 to 11-fold) (Figure 3C; Supplementary Table S4).

In contrast to the CaM-G114 variants, the Ca^2+^-dependent increase in FA_max_ observed for the CaM-WT/IQ-domain complex is also apparent for the CaM-I10T/IQ-domain complex (Figure 3B, *orange*). Like for CaM-WT, the FA_max_ value for the CaM-N98S/IQ-domain complex increases with Ca^2+^, but the transition is shifted to higher Ca^2+^-concentrations (Figure 3B, *green*, Supplementary Tables S1, S3). Further, the arrhythmogenic CaM-N98S variant only reduced the Na_V_1.5 affinity significantly (1.5-fold compared to CaM-WT) at intermediate 4 uM free Ca^2+^ (Figure 3C; Supplementary Tables S2, S4). This effect is consistent with the observed 4-fold reduction in CaM C-lobe Ca^2+^ affinity imposed by the N98S substitution, but different from the G114R and G114W substitutions, although they have a similar impact on C-lobe Ca^2+^ binding (3- and 7-fold reduction compared to CaM-WT) (Brohus et al., 2021). The non-arrhythmogenic CaM-I10T variant displayed no difference in IQ-domain affinity compared to CaM-WT across any of the Ca^2+^ concentrations tested (Figure3C, *orange*). These results are consistent with the observation that both residue I10 and N98 are located away from the CaM/Na_V_1.5 IQ-domain binding interface (Supplementary Figure S2).

The dramatic effect of the G114 variants on Na_V_1.5 affinity at low Ca^2+^ concentrations (≤200 nM) appear specific to the IQ-domain, as the effect of CaM-G114R and G114W on the Na_V_1.5 NTD were much smaller at the corresponding Ca^2+^ concentrations and comparable in magnitude to the effects of arrhythmogenic N98S (within a 4-fold difference from CaM-WT) (Supplementary Figure S3, S4; Supplementary Table S5, S6). As observed for the IQ-domain, the interaction between the non-arrhythmogenic CaM-I10T and the Na_V_1.5 NTD did not differ from the CaM-WT/NTD interaction.

## Discussion

In this study, we use an FA-based assay to investigate the Ca^2+^-dependent interactions between CaM-WT and the Na_V_1.5 IQ-domain and the recently identified CaM binding domain in the Na_V_1.5 NTD, and how these are affected by mutations in CaM.

Intriguingly, the CaM/Na_V_1.5 IQ-domain interaction displays a very different Ca^2+^ sensitivity profile compared to the interactions between CaM and the Na_V_1.5 NTD, Ca_V_1.2 IQ-domain, and RyR2-CaMBD2 (Wang et al., 2018; Brohus et al., 2019; Wang et al., 2020; Brohus et al., 2021). While the binding affinity increases 10-fold for the CaM-WT/Na_V_1.5 IQ-domain complex from low nM to high µM Ca^2+^-concentrations, the affinities of the CaM-WT/Na_V_1.5 NTD, CaM-WT/Ca_V_1.2 IQ-domain, and CaM-WT/RyR2-CaMBD2 complexes increase more than 1000-fold across the same Ca^2+^-range (Supplementary Figure S5) (Brohus et al., 2021). Moreover, apoCaM binds to the Na_V_1.5 IQ-domain with high affinity (117 nM—in good agreement with affinities determined by others (Shah et al., 2006; Yan et al., 2017)), much higher than to the Na_V_1.5 NTD (825-fold), the Ca_V_1.2 IQ-domain (26-fold), and RyR2 CaMBD2 (7-fold). For Ca^2+^-saturated CaM, the interaction with the Na_V_1.5 IQ-domain is more than 100-fold weaker than the interaction with the CaM binding domains from RyR2 and Ca_V_1.2 (Brohus et al., 2021). These results corroborate an essential role of apoCaM in modulating the Na_V_1.5 channel via the IQ-domain (Kang et al., 2021).

A potential dysregulation of Na_V_1.5, caused by human CaM mutations, has previously been investigated for a handful of LQTS-causing CaM variants (D96V, D130G, F142L, E141G) (Yin et al., 2014; Boczek et al., 2016; Rocchetti et al., 2017; Tarasov et al., 2023). However, the results for these variants have been largely unremarkable. Co-expression of CaM and human Na_V_1.5 in tsA102 cells, and subsequent whole-cell patch clamp recordings, showed no effect on channel function for any of the CaM variants investigated. Only when expressing a fetal Na_V_1.5 splice variant, CaM-D130G caused a 7.5-fold increase in persistent Na^+^ current, and only at 1 µM free Ca^2+^. Moreover, native Na^+^ currents from fetal mouse cardiomyocytes were not affected by CaM-D130G (Yin et al., 2014). Along the same lines, co-expression of CaM-E141G and Na_V_1.5 in tsA102 cells, and subsequent whole-cell patch clamp recordings, caused a 1.7-fold increase in persistent Na^+^ current, but the effect was no longer apparent when co-expressed with CaM-WT (Boczek et al., 2016). Since these studies, the Na_V_1.5 channel has been under the radar in terms of studying its implication in calmodulinopathies. However, and interestingly, Tarasov and co-workers recently demonstrated that CaM-D96V specifically increased the late current of the Na_V_1.6 isoform, but not of Na_V_1.5, speculating that this was due to a reduced CaM affinity for the Na_V_1.6 IQ-domain (Tarasov et al., 2023).

We have previously shown that CaM-G114R and -G114W reduce the affinity of CaM for both the Ca_V_1.2 IQ-domain and for RyR2-CaMBD2 at low to medium Ca^2+^-concentrations (Brohus et al., 2021). Here we show that both mutations also reduce CaM’s affinity for the Na_V_1.5 IQ-domain, but the effect is dramatically larger than for the CaMBDs of Ca_V_1.2 and RyR2, particularly at free Ca^2+^-concentrations ≤200 nM, the physiological Ca^2+^ concentration in the cardiomyocytes at rest, where the CaM/Na_V_1.5 IQ-domain interaction is essentially abolished.

The interaction between apoCaM and Na_V_1.5 is critical for channel function, by tuning channel activity. ApoCaM binding to the Na_V_1.5 CTD causes an increase in peak channel open probability as well as a decrease in persistent channel open probability, effects that have divergent implications in disease (Kang et al., 2021). Disruption or weakening of apoCaM binding reduces peak open probability of the channel, corresponding to a loss-of-function effect, such as that observed with the BrS phenotype. However, impaired apoCaM binding can also lead to an increase in persistent Na_V_1.5 late current, corresponding to a gain-of-function effect, such as that observed with the LQT3 phenotype (Yan et al., 2017; Kang et al., 2021). The dramatic reduction in the affinity of apoCaM for the Na_V_1.5 IQ-domain, caused by substitution of CaM-G114 could thus result in similar divergent effects, and may provide a mechanistic explanation for the mixed phenotypic pattern observed for carriers of CaM-G114 mutations, and potentially other residues affecting Na_V_1.5 binding.

But how can a mutation in one of six CaM-encoding alleles display a dominant effect through an ion-channel if the affinity for this channel is dramatically reduced? Several points provide hints towards a possible explanation. The intracellular pool of CaM is limited in cardiomyocytes, suggesting a dynamic competition among CaM target binding sites (Persechini and Stemmer, 2002; Wu et al., 2007). In the GTEx transcript database (GTEx Consortium, 2013), *CALM2* represents ∼50% of the total *CALM* transcript pool in ventricular tissue, and a heterozygous *CALM2* missense mutation may thus be present in 25% of the CaM protein pool. Since the impact of a specific CaM mutation differs between targets, the available CaM protein will be redistributed accordingly. Targets affected by a large reduction in CaM binding affinity may experience a larger deficit in CaM saturation than expected from the CaM-mutant/CaM-WT protein ratio, thereby increasing the risk of experiencing a “haploinsufficiency-like” phenotype. This notion is corroborated by a study investigating LQT3 mutations within the Na_V_1.5 IQ-domain (Yan et al., 2017). The study demonstrated that these variants increase the persistent Na^+^ current amplitude of Na_V_1.5 in whole-cell patch clamp recordings in HEK-cells, and that IQ-domains containing these mutations reduce the CaM binding affinity. Overexpression of CaM-WT rescued the increased current for these LQT3-Na_V_1.5 channels (Yan et al., 2017).

Another possible explanation for a dominant effect of the CaM-G114 variants is that CaM binds to other parts of the Na_V_1.5 channel than the IQ-domain. One example is binding of apoCaM to the preIQ-domain with high affinity (∼40 nM) (Yoder et al., 2019). Such binding may anchor the CaM-G114 variant to the channel and mediate a pathogenic effect through a compromised IQ-domain binding, induced by the C-lobe mutation.

Intrinsic mutations in Na_V_1.5 are responsible for BrS and LQTS3, arrhythmogenic conditions both known for cardiac events to frequently occur during rest/sleep (Schwartz et al., 2001; Postema and Wilde, 2008; Takigawa et al., 2008). Additionally, for BrS, fever has been established as a trigger of these events (Adler et al., 2013; Michowitz et al., 2018). Some of these arrhythmogenic channel mutations occur in the CaM-binding IQ-domain of the Na_V_1.5 CTD and perturb the CaM/Na_V_1.5 interaction (Supplementary Figure S1) (Shah et al., 2006; Yan et al., 2017; Kang et al., 2021; Wu and Liang, 2021). This, together with the impaired apoCaM/Na_V_1.5 IQ-domain interaction presented in this work, opens a possible mechanistic explanation for the clinical presentation observed for the CaM-G114R carriers, who died suddenly at a very young age while asleep, potentially triggered by a fever from the infections they each had at the time of death (Brohus et al., 2021). Other intrinsic Na_V_1.5 BrS/LQTS mutations occur in the NTD of the channel, a domain for which the role of CaM has only recently been explored (Wang et al., 2020). Wang and others demonstrated the ability of CaM to interact with the NTD of Na_V_1.5 and discussed the potential implication of altered CaM binding in the presence of intrinsic channel disease mutations. We find that the binding of CaM to the Na_V_1.5-NTD depends dramatically on Ca^2+^-concentration (Supplementary Figures S3, S4). When the Ca^2+^-concentration approaches µM range, the CaM/Na_V_1.5-NTD affinity increases and is comparable to that of the IQ-domain, supporting a potential Ca^2+^-triggered role of the Na_V_1.5 NTD in CaM regulation of channel activity.

It is not surprising that the phenotypic range of calmodulinopathies may not yet be fully mapped, as CaM interacts with a myriad of cardiac target proteins, that may or may not be affected by specific CaM missense mutations. In addition to CaM mutation effects on Na_V_1.5, evidence of K_V_7.1 effects are accumulating, further expanding the phenotypic spectrum of calmodulinopathies. Kato and others described a family of 14 CaM-N138K carriers who displayed a variably expressed LQTS phenotype from asymptomatic carriers to carriers experiencing sudden death as children (Kato et al., 2022). In support of the LQTS phenotype, the CaM-N138K variant caused an impairment of Ca_V_1.2 inactivation by whole-cell patch clamp recordings of HEK-cells. However, the variant also caused an unexpected potentiation of the K_V_7.1 current by the same technique in CHO cells, providing a possible explanation for the variably expressed LQTS phenotype, by countering the Ca_V_1.2 effects (Kato et al., 2022). Another comprehensive study, involving 13 arrhythmogenic CaM variants, revealed differential effects of the CaM variants on K_V_7.1 binding affinity, channel trafficking, and channel gating (activation) (Kang et al., 2023). Interestingly, as the only one of the 13 variants, CaM-G114W diminished the interaction with the K_V_7.1 channel, both at resting and elevated Ca^2+^ concentrations, and induced an increase in K_V_7.1 trafficking to the cell membrane (Kang et al., 2023).

**FIGURE 4:**
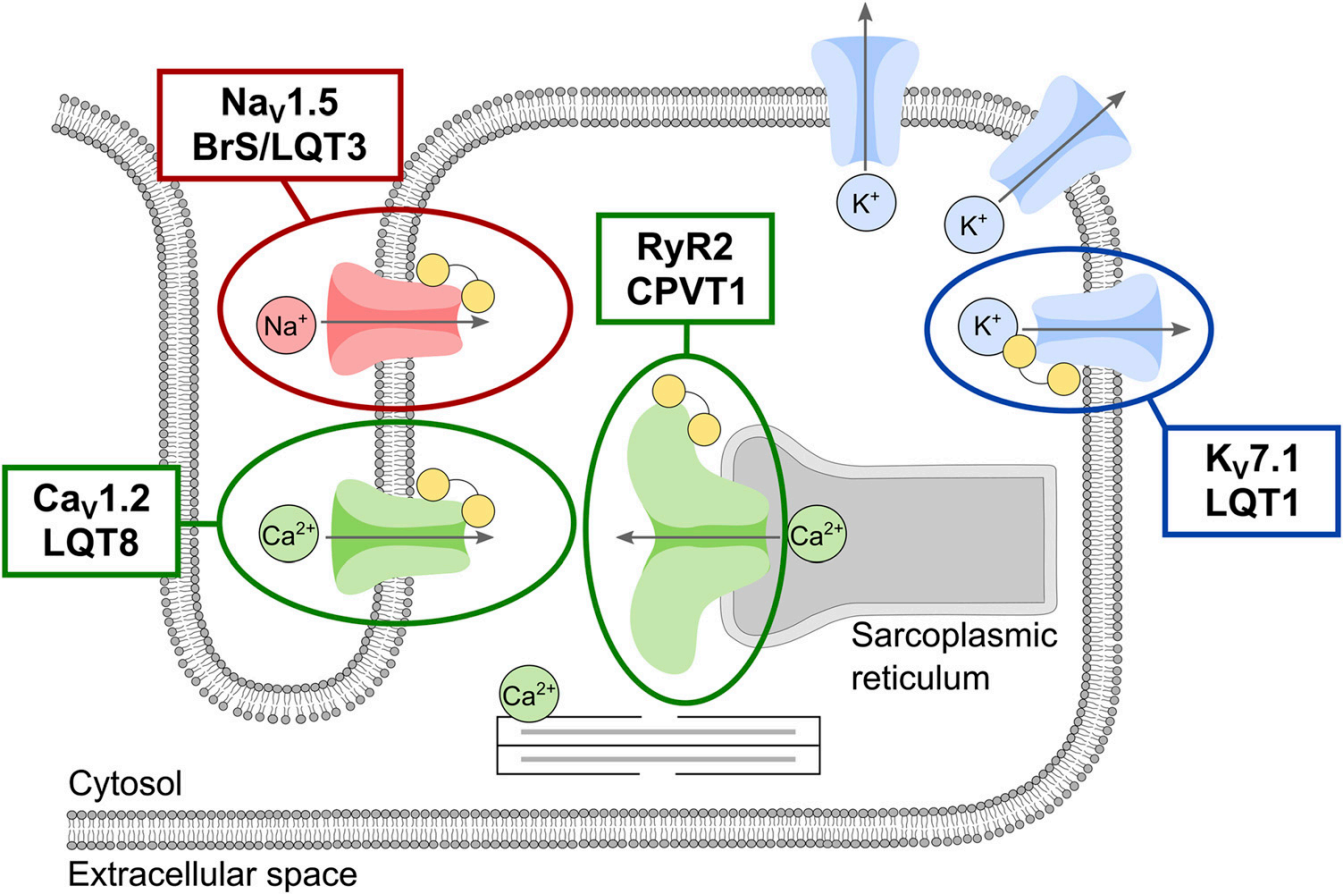
Schematic overview of the phenotypic spectrum of the main calmodulin (CaM)-regulated cardiac ion-channels responsible for the cardiac action potential and excitation-contraction coupling. Ion-channels are shown and colored according to the permeating ion: the ryanodine receptor (RyR2, *green*) and the voltage-gated calcium (Ca_V_1.2, *green*), sodium (Na_V_1.5, *red*), and potassium (K_V_7.1, *blue*) channels. CaM is shown as yellow dumbbells. The involvement of multiple potassium channels in shaping the cardiac action potential is represented by multiple copies of this channel. Clinical phenotypes associated with intrinsic channel mutations are given for the CaM-regulated channels.

In conclusion, the data presented in this study warrants a potential expansion of the phenotypic spectrum of calmodulinopathies. Moreover, these results emphasize our incomplete understanding of the molecular mechanisms possible for calmodulinopathy-related diseases and point to the complexity in variant interpreting due to the mixed phenotypes caused by individual CaM mutations. Molecularly, the multifaceted effects of CaM mutations may act additively or synergistically, thereby contributing to compound and mixed phenotypic expressions. Also, given the high number of CaM-binding targets in cardiomyocytes, the likelihood of observing variably expressed phenotypes in CaM mutation carriers increases, compared to carriers of single ion-channel (or single pathway effecting protein) mutations with pure ‘classical’ phenotypes (Figure 4). This brings the calmodulinopathies to the forefront of scientific research into personalized medicine. Careful interrogation of the *CALM* genes in large cohorts of sequenced individuals with unexplained BrS-like or atypical heart arrhythmia phenotypes should be performed to confirm an expansion of the phenotypic spectrum of calmodulinopathies to include *CALM*-BrS. Such knowledge will allow for earlier and more accurate diagnosis and treatment of individuals with calmodulinopathies.

## Data Availability

The original contributions presented in the study are included in the article/Supplementary Files, further inquiries can be directed to the corresponding author.
