# Supplementary material for: Calmodulin mutations affecting Gly114 impair binding to the NaV1.5 IQ-domain
Source: Front Pharmacol. 2023 Aug 16;14:1210140. doi: 10.3389/fphar.2023.1210140 (PMC10469309; doi:10.3389/fphar.2023.1210140)
Supplement: Supplementary file 1 [file DataSheet1.docx]

Supplementary Material

Calmodulin mutations affecting Gly114 impair binding to the NaV1.5 IQ-domain

Malene Brohus^1^, Ana-Octavia Busuioc^1^, Reinhard Wimmer^1^, Mette Nyegaard^2^, Michael Toft Overgaard^1*^

^1^Department of Chemistry and Bioscience, Aalborg University, Fredrik Bajers Vej 7H, 9220 Aalborg, Denmark

^2^Department of Health Science and Technology, Aalborg University, Selma Lagerløfs Vej 249, 9260 Gistrup, Denmark

*** Correspondence:**
Michael Toft Overgaard
[mto@bio.aau.dk](mailto:mto@bio.aau.dk)

# Supplementary Materials and Methods

## Protein alignment and structural representations

Alignment of human (UniProt ID: P0DP23), pig (UniProt ID: F2Z5G3), mouse (UniProt ID: P0DP26), zebrafish (UniProt ID: Q6PI52), fly (UniProt ID: P62152), worm (UniProt ID: O16305), plant (UniProt ID: P0DH95), and yeast CaM (UniProt ID: P06787) was done in CLC Main Workbench v. 8.1.2 using the very accurate alignment option with a gap open cost of 10 and a gap extension cost of 1.

Structural representations of CaM (PDB ID: 1CLL) and CaM/Na_V_1.5-CTD complexes (PDB IDs: 4JQ0, 4OVN, and 2L53) were prepared in the PyMol Molecular Graphics System v. 2.3.0 (Schrödinger, LLC).

## Protein expression and purification

Calmodulin variants were expressed as maltose-binding protein (MBP) fusion proteins from an IPTG-inducible pET vector in *E. coli* Rosetta (DE3). Following cell lysis by sonication, the MBP-CaM fusion proteins were purified by affinity chromatography using a custom packed amylose column (New England Biolabs) and an NGC Quest 100 Chromatography System (Bio-Rad Laboratories). The column was equilibrated with 3 column volumes (CV) of binding buffer (20 mM Tris, 100 mM NaCl, 1 mM EDTA, 1 mM DTT, pH 7.5) before applying the cell lysate after which the column was washed with binding buffer until a stable UV baseline was reached. The fusion proteins were eluted by supplementing the binding buffer with 10 mM maltose.

Following affinity chromatography, the fusion proteins were cleaved by Tobacco Etch Virus (TEV) protease (enzyme:substrate 1:100 w:w) overnight at 4°C to separate CaM from MBP. The cleaved sample was applied to a custom packed Q Sepharose FF anion exchange column (Cytiva) equilibrated with binding buffer (20 mM Tris, 100 mM NaCl, pH 7.4) using an ÄKTA Purifier Chromatography System (Cytiva). The column was washed with 4 CV 40% elution buffer (20 mM Tris, 500 mM NaCl, pH 7.4) after which the proteins were eluted by applying a linear gradient of 40-90% elution buffer over 7 CV followed by a 2 CV wash with 100% elution buffer.

The CaM-containing fractions were pooled and concentrated using a 10 MWCO Amicon centrifugal filter (Merck Millipore). 20 mM EDTA was added to the concentrated protein sample to remove residual Ca^2+^ before applying the sample to a HiLoad 16/600 Superdex 75 pg size exclusion column (Cytiva) equilibrated with buffer (20 mM HEPES, 100 mM KCl, pH 7.2) using an ÄKTA Purifier Chromatography System (Cytiva). The CaM-containing fractions were pooled, and the identity of each CaM variant was confirmed by MALDI-TOF analysis of both intact and tryptic digested proteins using an autoflex speed mass spectrometer (Bruker) in linear positive (LP) and reflector positive (RN) mode, respectively. During the purification process, the presence and purity of the protein was evaluated by reducing SDS-PAGE using a 4-20% SurePAGE Bis-Tris gel (Genscript).

## Ca/Mg buffers

Assay buffers of varying free concentrations of Ca^2+^, in the presence of 1 mM free Mg^2+^, were prepared using a chelator system previously described (Dweck, Reyes-Alfonso, and Potter 2005). Based on the chemical composition of the buffer (50 mM HEPES, 100 mM KCl, 0.5 mM EGTA, 2 mM NTA, pH 7.2), a pCa calculator was used to determine the concentrations of total Ca^2+^ required to obtain a range of free Ca^2+^ concentrations. In practice, 1.5x concentrated buffers were prepared to be able to include the CaMBD and CaM during dilution to 1x. Eight Ca^2+^-buffered solutions (0.3 nM – 400 µM free Ca^2+^) were prepared by mixing appropriate volumes of three 1.5x buffers containing no Ca^2+^, 30 mM Mg^2+^, or 3 mM Ca^2+^, respectively.

## Na_V_1.5-IQ binding domain

Lyophilized TAMRA-labeled Na_V_1.5 IQ-domain (5-TAMRA-EEVSAMVIQRAFRRHLLQRSLKHASFL-NH_2_, purity > 95%) and Na_V_1.5 NTD (5-TAMRA- TIFRFSATNALYVLSPFHPIRRAAVK-NH_2_, purity > 95%) was purchased from Proteogenix. The peptides were dissolved in 5% acetonitrile/0.1% TFA and the concentration was determined by 5-TAMRA absorbance at 556 nm (Ɛ = 90,000 M^-1^cm^-1^). The purity and integrity of the peptides was assessed by LC-MS using a hexyl-phenyl column attached to an Elite LaChrom HPLC system (Hitachi) and a Compact qTOF mass spectrometer (Bruker).

## CaM/Na_V_1.5 IQ-domain binding assay

The interaction between CaM and the TAMRA-labeled Na_V_1.5 domains was measured using a previously described plate-based assay monitoring fluorescence anisotropy (FA) (Brohus et al. 2019). In this assay, the total concentration of CaM was varied in one dimension (across columns) and the free concentration of Ca^2+^ in the other (across rows) using a microlab STARlet liquid handling robot (Hamilton).

The FA signal from the 5-TAMRA labelled IQ-domain or NTD was measured in a Tecan SPARK plate reader using a 535(25) nm excitation filter, a 590(20) nm emission filter, and a dichroic 560 mirror. The signal was acquired using 20 flashes and a settle time of 200 ms. The G-factor was measured with free 5-TAMRA and set to 0.990.

## Curve fitting and statistical testing

A 1:1 binding model was fitted to the data resulting from the binding assay (see above), by non-linear curve fitting using GraphPad Prism v. 6.07:

$$Y=\frac{K_{D}+\left[ CaMBD \right]_{tot}+{[CaM]}_{tot}}{2\cdot{[CaMBD]}_{tot}}-\sqrt{\left( \frac{K_{D}+\left[ CaMBD \right]_{tot}+{[CaM]}_{tot}}{2\cdot{[CaMBD]}_{tot}} \right)^{2}-\frac{{[CaM]}_{tot}}{{[CaMBD]}_{tot}}}$$

Where Y denotes the fractional saturation of CaMBD with CaM, [CaMBD]_tot_ and [CaM]_tot_ are the total concentrations of CaMBD peptide and CaM, respectively, and K_D_ is the dissociation constant of the CaMBD/CaM interaction.

The monitored FA signal reflects the transition from free CaMBD (FA_CaMBD_) to CaMBD saturated with CaM (FA_CaMBD/CaM_):

$$FA={FA}_{CaMBD}\cdot\left( 1-Y \right)+{FA}_{CaMBD/CaM}\cdot Y$$

Data fitting was done by combining the two equations and solving for FA.

At low Ca^2+^-concentrations, the binding curves for CaM-G114R and -G114W (IQ-domain) or all CaM variants (NTD) did not reach an upper FA plateau. For these data, K_D_ values were estimated by assuming a similar value for the upper FA plateau as that observed at higher Ca^2+^-concentrations.

Statistical testing of differences in FA_max_ and K_D_ values between CaM-WT and variants was done using a 1-way ANOVA on non-transformed and log10-transformed FA values, respectively, at each free Ca^2+^-concentration. Dunnett’s post hoc test was used to correct for multiple comparisons. Significance levels were indicated by asterisks: ******** (P-value < 0.0001), *** (P-value < 0.001), * (P-value < 0.05).

# Supplementary Figures and Tables

## Supplementary Figures


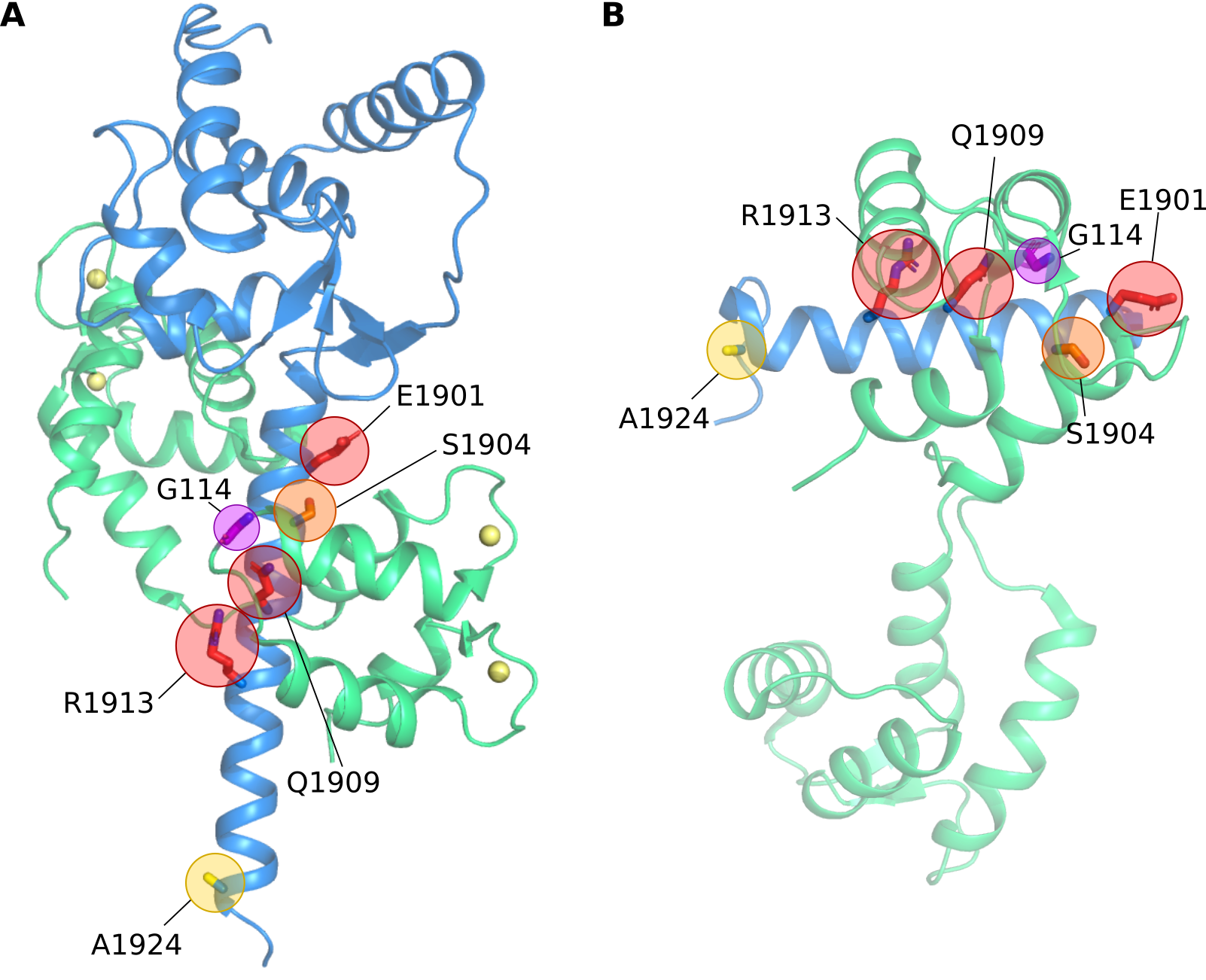


Supplementary Figure 1: Visualization of Na_V_1.5 IQ-domain missense variants associated with LQT3 (*red*), BrS (*yellow*) or both phenotypes (*orange*) (Kapplinger et al. 2010, 2015; Napolitano et al. 2005; Bankston et al. 2007; Kapplinger et al. 2009; Kang et al. 2021; Wu and Hong 2021), in a complex with Mg^2+^CaM (A, PDB ID 4OVN) (Gabelli et al. 2014) or apoCaM (B, PDB ID 2L53) (Chagot and Chazin 2011). Na_V_1.5 (*blue*) and CaM (*green*) are shown in cartoon representation. CaM-G114 (*purple*) is shown in stick representation. The Ca^2+^CaM/Na_V_1.5-CTD complex is not shown since the electron density of the amino acid side chains in most of the IQ-domain is lacking. Na_V_1.5 residue numbering is given according to transcript reference NM_000335.5. Na_V_1.5, voltage-gated sodium channel type 1.5; LQT3, long-QT syndrome type 3; BrS, Brugada syndrome; CaM, calmodulin.


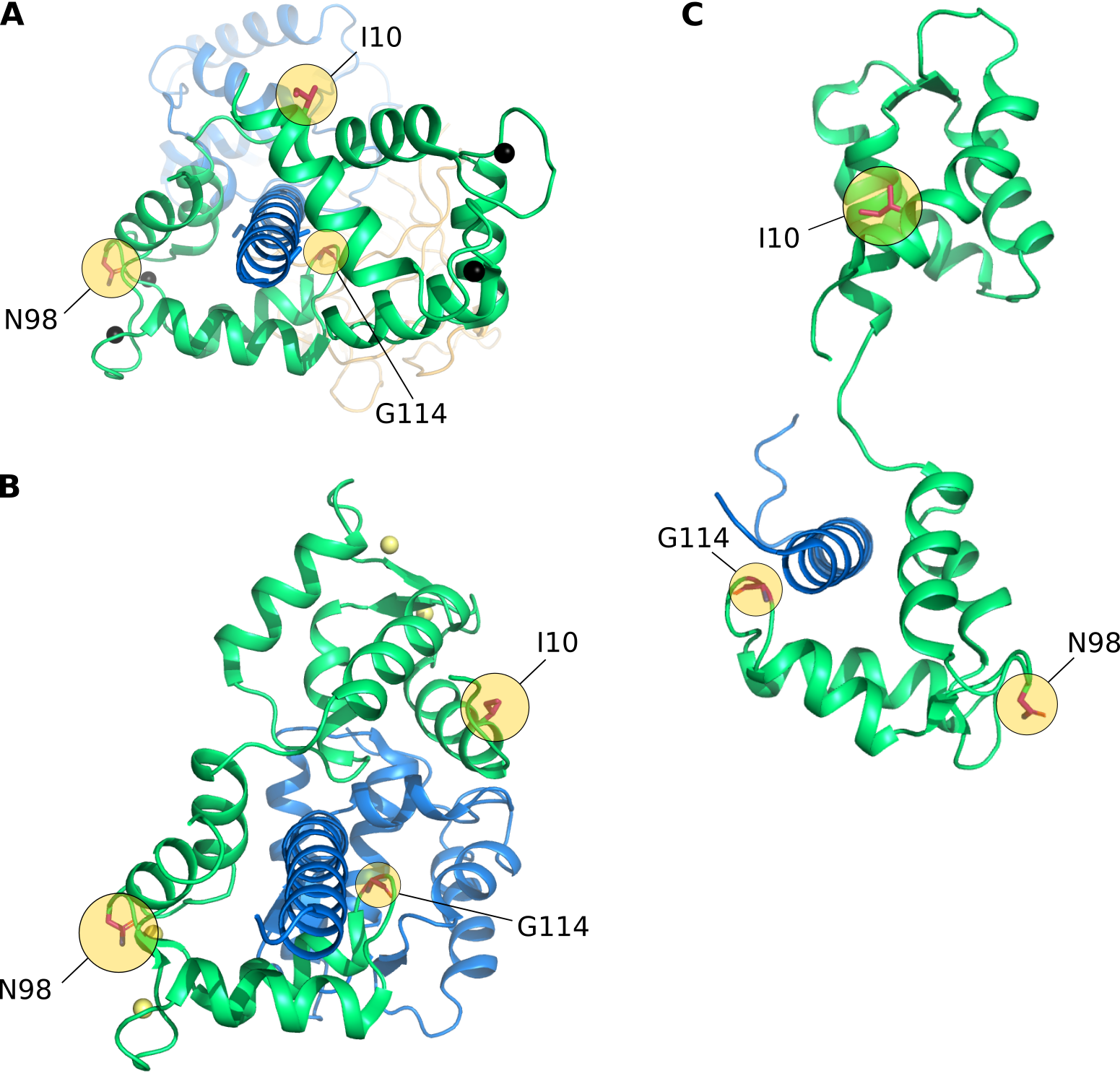


Supplementary Figure 2: Visualization of CaM-I10, -N98, and -G114 in complexes with the Na_V_1.5 CTD (A and B, PDB IDs: 4OVN (Gabelli et al. 2014) and 4JQ0 (Wang et al. 2014)) or IQ-domain (C, PDB ID: 2L53 (Chagot and Chazin 2011)). CaM (*green*) and Na_V_1.5 domains (*blue*) are shown in cartoon representation, and Ca^2+^ (*black*) and Mg^2+^ (*yellow*) ions are shown as spheres.


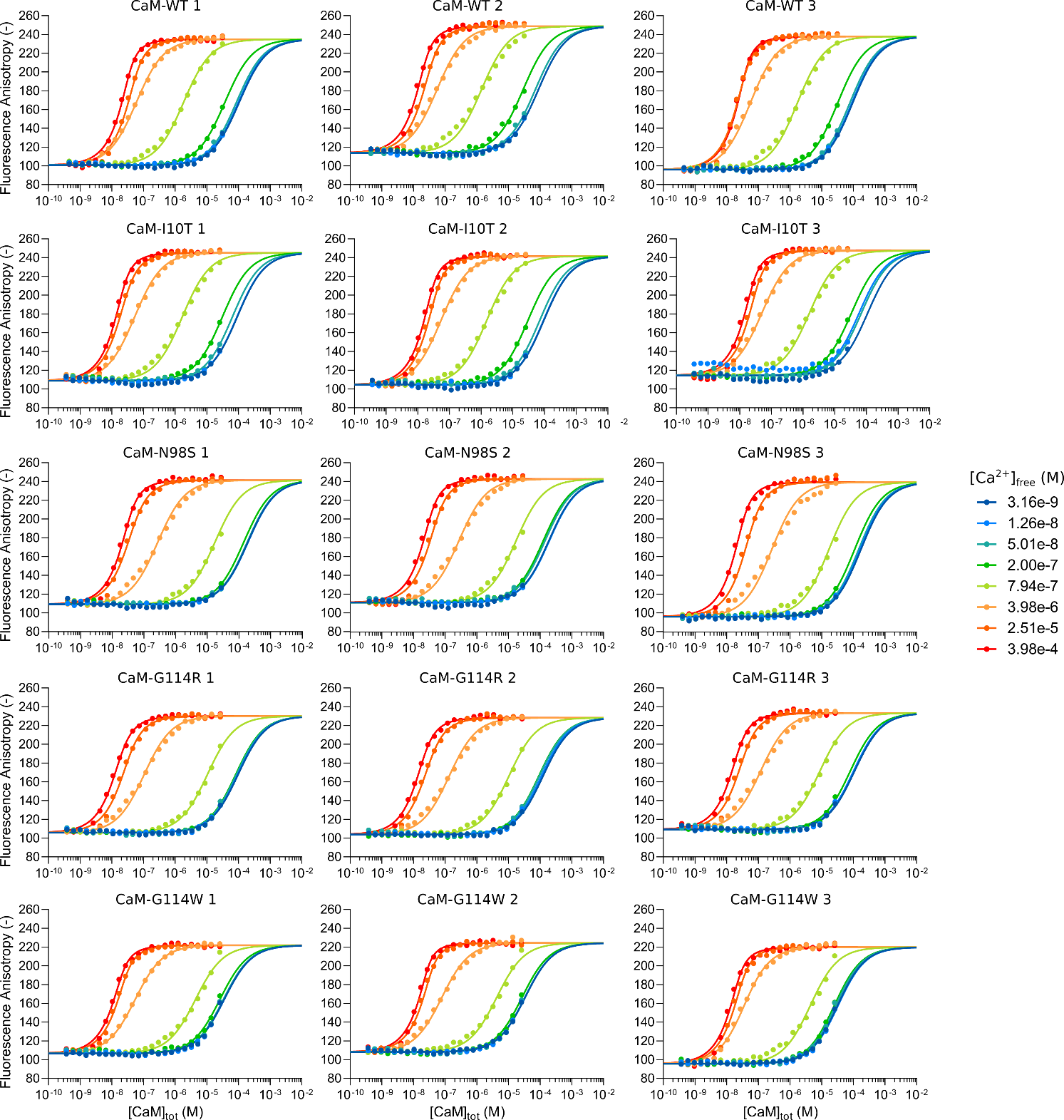


Supplementary Figure 3: Calmodulin (CaM) binding to the Na_V_1.5 N-terminal domain (NTD) monitored by fluorescence anisotropy (FA) as a function of total CaM-concentration ([CaM]_tot_) at eight free Ca^2+^ concentrations ([Ca^2+^]_free_). Each panel represents individual replicates and curves represent best fits based on a single site binding model. At low [Ca^2+^]_free_, where an upper plateau was not reached, the assumption was made that the plateau was the same as at high [Ca^2+^]_free_ (extrapolated curve fit).


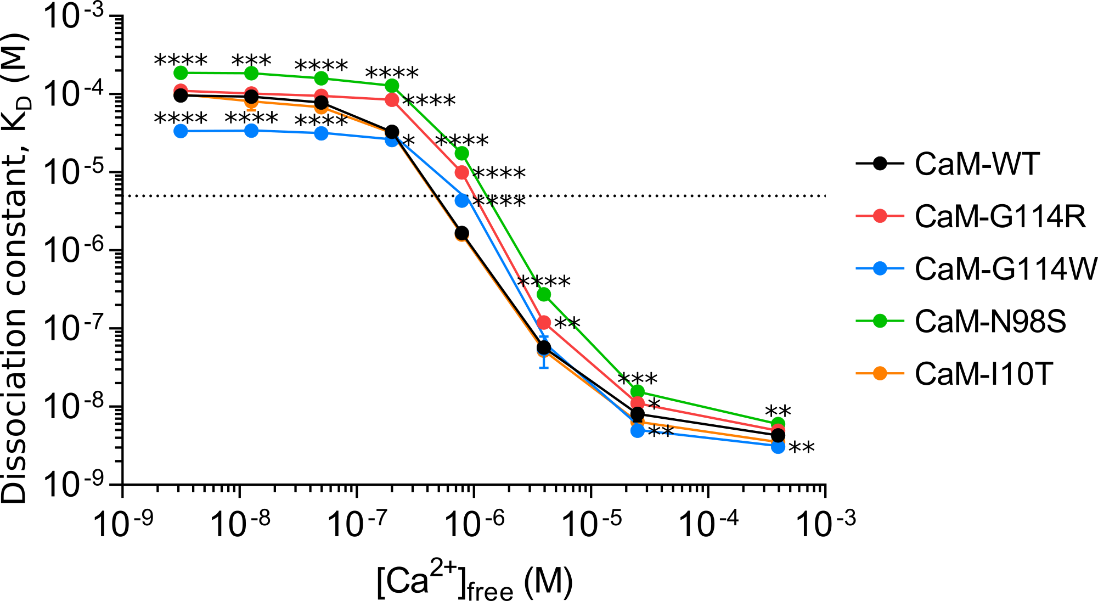


Supplementary Figure 4: Ca^2+^-dependent changes in the CaM affinity of the Na_V_1.5 N-terminal domain (NTD) represented by the dissociation constant (K_D_) as a function of [Ca^2+^]_free_. Each data point represents the mean of three replicates with the standard deviation shown as error bars. Statistically significant differences between CaM-WT and other variants were determined by a 1-way ANOVA at each Ca^2+^-concentration with Dunnett’s multiple comparisons test: **** (P-value < 0.0001), *** (P-value < 0.001), * (P-value < 0.05).


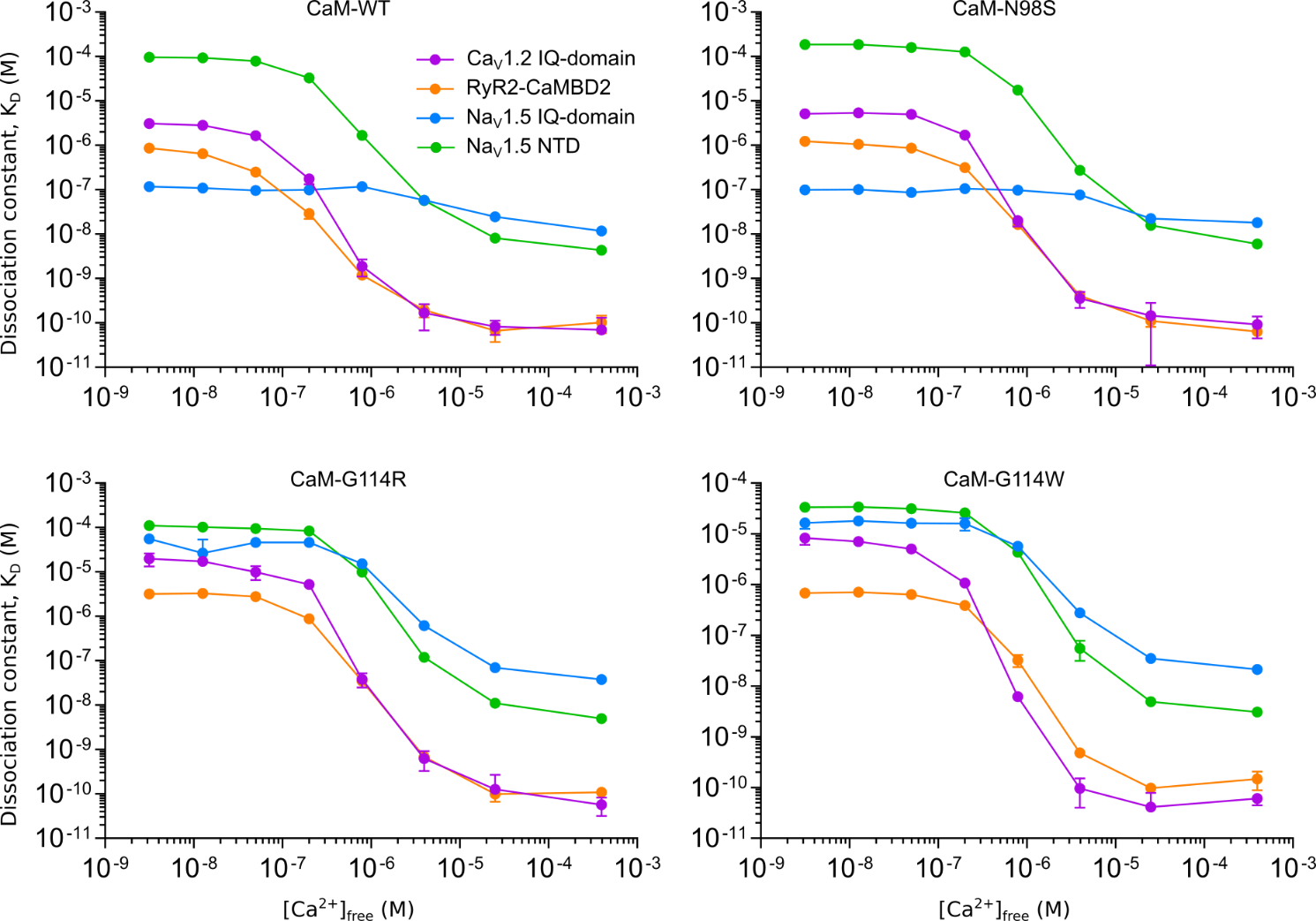


Supplementary Figure 5: Calcium-dependent binding of calmodulin (CaM) variants to the primary CaM-binding domains (CaMBDs) of cardiac ion channels: the CaMBD2 of the ryanodine receptor 2 (RyR2, *orange*), the IQ-domain (*blue*) and the N-terminal domain (NTD, *green*) of the voltage-gated sodium channel (Na_V_1.5), and the IQ-domain of the voltage-gated calcium channel (Ca_V_1.2, *purple*). Data for RyR2 and Ca_V_1.2 are from our previous study (Brohus et al. 2021).

## Supplementary Tables

Supplementary Table 1: Maximum fluorescence anisotropy (FA_max_) values of the interaction between CaM and the Na_V_1.5 IQ-domain at different free Ca^2+^ concentrations. Values represent the mean ± standard deviation (SD) of three experimental replicates. Bolded values indicate significantly different FA_max_ values compared to CaM-WT (P-value < 0.05).

|  | CaM-WT | | CaM-I10T | | CaM-N98S | | CaM-G114R | | CaM-G114W | |
| --- | --- | --- | --- | --- | --- | --- | --- | --- | --- | --- |
| [Ca^2+^]_free_ | FA_max_ | SD | FA_max_ | SD | FA_max_ | SD | FA_max_ | SD | FA_max_ | SD |
| 3 nM | 136.0 | *1.1* | 135.1 | *1.7* | 133.3 | *1.4* | **-** | - | **-** | - |
| 13 nM | 136.4 | *2.3* | 135.1 | *2.1* | 133.6 | *1.5* | **-** | - | **-** | - |
| 50 nM | 137.1 | *2.0* | 137.1 | *2.3* | 133.2 | *1.0* | **-** | - | **-** | - |
| 200 nM | 138.5 | *1.3* | 138.4 | *1.4* | **134.7** | *1.5* | **-** | - | **-** | - |
| 800 nM | 144.9 | *1.3* | 145.3 | *1.5* | **135.9** | *1.7* | **-** | - | **-** | - |
| 4 µM | 159.9 | *1.9* | 162.3 | *2.1* | **145.0** | *1.7* | **190.6** | *0.9* | **168.5** | *0.8* |
| 25 µM | 180.7 | *0.2* | 182.6 | *1.3* | **166.0** | *1.2* | **198.8** | *0.5* | **168.7** | *0.7* |
| 400 µM | 182.6 | *1.5* | 185.3 | *0.8* | **177.8** | *1.9* | **199.2** | *0.9* | **166.9** | *0.6* |

Supplementary Table 2: Dissociation constants (K_D_) of the interaction between CaM and the Na_V_1.5 IQ-domain at different free Ca^2+^ concentrations. Values represent the mean ± standard deviation (SD) of three experimental replicates. Bolded values indicate significantly different affinities compared to CaM-WT (P-value < 0.05).

|  | CaM-WT | | CaM-I10T | | CaM-N98S | | CaM-G114R | | CaM-G114W | |
| --- | --- | --- | --- | --- | --- | --- | --- | --- | --- | --- |
| [Ca^2+^]_free_ | K_D_ (nM) | SD (nM) | K_D_ (nM) | SD (nM) | K_D_ (nM) | SD (nM) | K_D_ (nM) | SD (nM) | K_D_ (nM) | SD (nM) |
| 3 nM | 117 | *17* | 124 | *11* | 99 | *15* | **55,080** | *8,226* | **16,407** | *3,845* |
| 13 nM | 109 | *15* | 116 | *12* | 100 | *11* | **26,442** | *27,378* | **18,103** | *1,902* |
| 50 nM | 97 | *8* | 110 | *33* | 86 | *15* | **46,180** | *8,611* | **16,117** | *2,624* |
| 200 nM | 99 | *21* | 132 | *38* | 105 | *21* | **45,737** | *1,680* | **15,907** | *4,355* |
| 800 nM | 117 | *5* | 135 | *48* | 98 | *22* | **15,207** | *475* | **5,719** | *667* |
| 4 µM | 58 | *4* | 51 | *6* | **76** | *11* | **612** | *7* | **279** | *18* |
| 25 µM | 24 | *3* | 20 | *4* | 22 | *2* | **69** | *4* | **35** | *3* |
| 400 µM | 12 | *2* | 16 | *6* | 18 | *1* | **38** | *6* | **21** | *4* |

Supplementary Table 3: Changes in the maximum fluorescence anisotropy (FA_max_) of CaM/Na_V_1.5 IQ-domain complexes given as the difference between CaM-WT and disease-variants. Bolded values indicate significantly different FA_max_ values compared to CaM-WT (P-value < 0.05).

| [Ca^2+^]_free_ | CaM-I10T (FA_max,Mut_ - FA_max,WT_) | CaM-N98S (FA_max,Mut_ - FA_max,WT_) | CaM-G114R (FA_max,Mut_ - FA_max,WT_) | CaM-G114W (FA_max,Mut_ - FA_max,WT_) |
| --- | --- | --- | --- | --- |
| 3 nM | -0.9 | -2.7 | - | - |
| 13 nM | -1.3 | -2.8 | - | - |
| 50 nM | 0.1 | -3.9 | - | - |
| 200 nM | 0.0 | **-3.8** | - | - |
| 800 nM | 0.4 | **-9.0** | - | - |
| 4 µM | 2.4 | **-14.9** | **-30.7** | **-8.6** |
| 25 µM | 1.9 | **-14.7** | **-18.1** | **12.0** |
| 400 µM | 2.7 | **-4.8** | **-16.6** | **15.7** |

Supplementary Table 4: Changes in CaM/Na_V_1.5 IQ-domain dissociation constants (K_D_) given as the ratio between disease-variants and CaM-WT. Bolded values indicate significantly different affinities compared to CaM-WT (P-value < 0.05).

| [Ca^2+^]_free_ | CaM-I10T (K_D,Mut_/K_D,WT_) | CaM-N98S (K_D,Mut_/K_D,WT_) | CaM-G114R (K_D,Mut_/K_D,WT_) | CaM-G114W (K_D,Mut_/K_D,WT_) |
| --- | --- | --- | --- | --- |
| 3 nM | 1.1 | 0.8 | **470.6** | **140.2** |
| 13 nM | 1.1 | 0.9 | **243.6** | **166.8** |
| 50 nM | 1.1 | 0.9 | **478.4** | **167.0** |
| 200 nM | 1.3 | 1.1 | **462.8** | **161.0** |
| 800 nM | 1.2 | 0.8 | **130.0** | **48.9** |
| 4 µM | 0.9 | **1.3** | **10.6** | **4.8** |
| 25 µM | 0.8 | 0.9 | **2.8** | **1.4** |
| 400 µM | 1.4 | 1.5 | **3.2** | **1.8** |

Supplementary Table 5: Dissociation constants (K_D_) of the interaction between CaM and the Na_V_1.5 N-terminal domain (NTD) at different free Ca^2+^ concentrations. Values represent the mean ± standard deviation (SD) of three experimental replicates. Bolded values indicate significantly different affinities compared to CaM-WT (P-value < 0.05).

|  | CaM-WT | | CaM-I10T | | CaM-N98S | | CaM-G114R | | CaM-G114W | |
| --- | --- | --- | --- | --- | --- | --- | --- | --- | --- | --- |
| [Ca^2+^]_free_ | K_D_ (nM) | SD (nM) | K_D_ (nM) | SD (nM) | K_D_ (nM) | SD (nM) | K_D_ (nM) | SD (nM) | K_D_ (nM) | SD (nM) |
| 3 nM | 96,543 | *9,000* | 110,307 | *12,620* | **33,613** | *1,854* | 99,073 | *9,513* | **186,733** | *11,637* |
| 13 nM | 92,623 | *6,416* | 101,657 | *7,218* | **34,030** | *1,447* | 80,773 | *18,763* | **185,833** | *20,823* |
| 50 nM | 78,233 | *8,115* | 94,573 | *7,670* | **31,330** | *3,109* | 68,333 | *3,168* | **160,167** | *25,466* |
| 200 nM | 32,863 | *4,233* | 84,363 | *5,559* | **25,983** | *760* | **31,690** | *1,912* | **127,567** | *18,387* |
| 800 nM | 1,661 | *230* | 9,895 | *949* | **4,359** | *99* | **1,595** | *97* | **17,520** | *547* |
| 4 µM | 57 | *3* | 120 | *14* | **55** | *24* | **52** | *2* | 275 | *8* |
| 25 µM | 8 | *2* | 11 | *0* | **5** | *0* | **6** | *1* | **16** | *2* |
| 400 µM | 4 | *1* | 5 | *0* | **3** | *0* | 4 | *0* | **6** | *1* |

Supplementary Table 6: Changes in CaM/Na_V_1.5-NTD dissociation constants (K_D_) given as the ratio between disease-variants and CaM-WT. Bolded values indicate significantly different affinities compared to CaM-WT (P-value < 0.05).

| [Ca^2+^]_free_ | CaM-I10T (K_D,Mut_/K_D,WT_) | CaM-N98S (K_D,Mut_/K_D,WT_) | CaM-G114R (K_D,Mut_/K_D,WT_) | CaM-G114W (K_D,Mut_/K_D,WT_) |
| --- | --- | --- | --- | --- |
| 3 nM | 1.14 | **0.35** | 1.03 | **1.93** |
| 13 nM | 1.10 | **0.37** | 0.87 | **2.01** |
| 50 nM | 1.21 | **0.40** | 0.87 | **2.05** |
| 200 nM | 2.57 | **0.79** | **0.96** | **3.88** |
| 800 nM | 5.96 | **2.62** | **0.96** | **10.55** |
| 4 µM | 2.09 | **0.96** | **0.92** | 4.80 |
| 25 µM | 1.35 | **0.61** | **0.79** | **1.93** |
| 400 µM | 1.15 | **0.71** | 0.82 | **1.40** |

# Supplementary References

Bankston, John R., Kevin J. Sampson, Suneel Kateriya, Ian W. Glaaser, David L. Malito, Wendy K. Chung, and Robert S. Kass. 2007. “A Novel LQT-3 Mutation Disrupts an Inactivation Gate Complex with Distinct Rate-Dependent Phenotypic Consequences.” *Channels (Austin, Tex.)* 1 (4): 273–80. https://doi.org/10.4161/chan.4956.

Brohus, Malene, Todor Arsov, David A Wallace, Helene Halkjær Jensen, Mette Nyegaard, Lia Crotti, Marcin Adamski, et al. 2021. “Infanticide vs. Inherited Cardiac Arrhythmias.” *Europace* 23 (3): 441–50. https://doi.org/10.1093/europace/euaa272.

Brohus, Malene, Mads T. Søndergaard, Sui Rong Wayne Chen, Filip van Petegem, and Michael T. Overgaard. 2019. “Ca2+-Dependent Calmodulin Binding to Cardiac Ryanodine Receptor (RyR2) Calmodulin-Binding Domains.” *The Biochemical Journal* 476 (2): 193–209. https://doi.org/10.1042/BCJ20180545.

Chagot, Benjamin, and Walter J. Chazin. 2011. “Solution NMR Structure of Apo-Calmodulin in Complex with the IQ Motif of Human Cardiac Sodium Channel NaV1.5.” *Journal of Molecular Biology* 406 (1): 106–19. https://doi.org/10.1016/j.jmb.2010.11.046.

Dweck, David, Avelino Reyes-Alfonso, and James D. Potter. 2005. “Expanding the Range of Free Calcium Regulation in Biological Solutions.” *Analytical Biochemistry* 347 (2): 303–15. https://doi.org/10.1016/j.ab.2005.09.025.

Gabelli, Sandra B., Agedi Boto, Victoria Halperin Kuhns, Mario A. Bianchet, Federica Farinelli, Srinivas Aripirala, Jesse Yoder, Jean Jakoncic, Gordon F. Tomaselli, and L. Mario Amzel. 2014. “Regulation of the NaV1.5 Cytoplasmic Domain by Calmodulin.” *Nature Communications* 5 (November): 5126. https://doi.org/10.1038/ncomms6126.

Kang, Po Wei, Nourdine Chakouri, Johanna Diaz, Gordon F. Tomaselli, David T. Yue, and Manu Ben-Johny. 2021. “Elementary Mechanisms of Calmodulin Regulation of NaV1.5 Producing Divergent Arrhythmogenic Phenotypes.” *Proceedings of the National Academy of Sciences of the United States of America* 118 (21): e2025085118. https://doi.org/10.1073/pnas.2025085118.

Kapplinger, Jamie D., John R. Giudicessi, Dan Ye, David J. Tester, Thomas E. Callis, Carmen R. Valdivia, Jonathan C. Makielski, Arthur A. Wilde, and Michael J. Ackerman. 2015. “Enhanced Classification of Brugada Syndrome-Associated and Long-QT Syndrome-Associated Genetic Variants in the SCN5A-Encoded Na(v)1.5 Cardiac Sodium Channel.” *Circulation. Cardiovascular Genetics* 8 (4): 582–95. https://doi.org/10.1161/CIRCGENETICS.114.000831.

Kapplinger, Jamie D., David J. Tester, Marielle Alders, Begoña Benito, Myriam Berthet, Josep Brugada, Pedro Brugada, et al. 2010. “An International Compendium of Mutations in the SCN5A-Encoded Cardiac Sodium Channel in Patients Referred for Brugada Syndrome Genetic Testing.” *Heart Rhythm* 7 (1): 33–46. https://doi.org/10.1016/j.hrthm.2009.09.069.

Kapplinger, Jamie D., David J. Tester, Benjamin A. Salisbury, Janet L. Carr, Carole Harris-Kerr, Guido D. Pollevick, Arthur A M Wilde, and Michael J. Ackerman. 2009. “Spectrum and Prevalence of Mutations from the First 2,500 Consecutive Unrelated Patients Referred for the FAMILION Long QT Syndrome Genetic Test.” *Heart Rhythm* 6 (9): 1297–1303. https://doi.org/10.1016/j.hrthm.2009.05.021.

Napolitano, Carlo, Silvia G. Priori, Peter J. Schwartz, Raffaella Bloise, Elena Ronchetti, Janni Nastoli, Georgia Bottelli, Marina Cerrone, and Sergio Leonardi. 2005. “Genetic Testing in the Long QT Syndrome: Development and Validation of an Efficient Approach to Genotyping in Clinical Practice.” *JAMA* 294 (23): 2975–80. https://doi.org/10.1001/jama.294.23.2975.

Wang, Chaojian, Ben C. Chung, Haidun Yan, Hong-Gang Wang, Seok-Yong Lee, and Geoffrey S. Pitt. 2014. “Structural Analyses of Ca^2+^/CaM Interaction with NaV Channel C-Termini Reveal Mechanisms of Calcium-Dependent Regulation.” *Nature Communications* 5 (1): 4896. https://doi.org/10.1038/ncomms5896.

Wu, Xin, and Liang Hong. 2021. “Calmodulin Interactions with Voltage-Gated Sodium Channels.” *International Journal of Molecular Sciences* 22 (18). https://doi.org/10.3390/ijms22189798.
